# Two kinds of common prenatal screening tests for Down’s syndrome: a systematic review and meta-analysis

**DOI:** 10.1038/srep18866

**Published:** 2016-01-06

**Authors:** Yuan Yao, Yang Liao, Mei Han, Sheng-Lan Li, Juan Luo, Bo Zhang

**Affiliations:** 1 Department of Laboratory Medicine, Southwest Hospital, Third Military Medical University of PLA, Chongqing 400038, PR China; 2 Department of Laboratory Medicine, No. 191 Clinical Department of No. 303 Hospital of PLA, Guigang 537100, Guangxi, PR China; 3 Department of Laboratory Medicine, Guangzhou General Hospital of Guangzhou Military Command of PLA, Guangzhou 510010, Guangdong, PR China

## Abstract

As the chromosomal examination of foetal cells for the prenatal diagnosis of Down’s syndrome (DS) carries a risk of inducing miscarriage, serum screening tests are commonly used before invasive procedures. In this study, a total of 374 records from PubMed, EMBASE, and the ISI Science Citation Index databases were reviewed. As a result of duplication, insufficient data, and inappropriate article types, 18 independent articles containing 183,998 samples were used in the final systematic review and meta-analysis of the diagnostic performance of the serum triple screening test (STS) and the integrated screening test (INS). Data extracted from the selected studies were statistically analysed, and the presence of heterogeneity and publication bias was assessed using specific software. The overall sensitivity, specificity, positive likelihood ratio, negative likelihood ratio, diagnostic odds ratio, and the area under the curve for the STS were 0.77 (95% confidence interval = 0.73–0.81), 0.94 (0.94–0.94), 9.78 (6.87–13.93), 0.26 (0.22–0.31), 44.72 (30.77–65.01), and 0.9064, respectively. For the INS, these values were 0.93 (0.90–0.95), 0.93 (0.93–0.93), 22.38 (12.47–40.14), 0.08 (0.05–0.11), 289.81 (169.08–496.76), and 0.9781, respectively. These results indicate that the INS exhibits better diagnostic value for DS. However, further research is needed to identify other biomarkers to improve prenatal screening tests.

Down’s syndrome (DS), also known as trisomy 21, is one of the most common congenital developmental disabilities caused by chromosomal disorders in humans^1^,^2^, with a morbidity of 1 in 600–800 newborn infants^3^. The majority of patients with DS have standard trisomy 21, a condition in which an entire extra chromosome 21 exists in all cells; the other patients with DS have mosaics or translocations^4^. DS individuals exhibit various clinical symptoms in which multiple organs and physiological systems are involved. Cognitive disability and impaired social adaptability from birth are quite common in individuals with DS^5^, and interrelated physical impairment and disability, including dementia, gastrointestinal complications, thyroid disorder, and so on, are also universal in most DS-affected infants^6^,^7^,^8^. However, only approximately half of DS-affected patients suffer from congenital cardiovascular defects^5^. Recently, the incidence of DS has been increasing due to many physiological and social factors, for example, rising maternal ages^9^. Patients with DS are usually deprived of self-care agency, which may lead to problems for the patients’ families and for society in general. Therefore, prenatal screening and effective diagnostics are needed to evaluate the risk of a DS-affected pregnancy^10^,^11^ and to provide more choices for pregnant women^3^. Non-invasive prenatal testing (NIPT) using cell-free foetal DNA or cell-free foetal placental-specific mRNA in maternal plasma is a very effective prenatal screening tool for DS. Recently, the utility of this prenatal test for the screening of DS and other genetic conditions has been extensively studied^12^,^13^. Studies have reported sensitivities of 98–100% and specificities of 97–100% for NIPT^14^. This test has resulted in a 95% decrease in the number of invasive procedures performed on pregnant women and a 99% decrease in the number of unaffected pregnancy losses^15^, which suggests that NIPT is a highly effective tool for prenatal DS screening. However, NIPT cannot be used in all pregnancies because of its high cost and the significant amount of time required to perform the test.

Consequently, the most commonly used prenatal DS screening strategy that can be used on a large-scale is based on predicting risk using a combination of gestational age, maternal age and weight, maternal biochemical markers, and ultrasound measurements. Currently, multiple-marker prenatal screening for DS has become an established practice in most countries. These voluntary screening tests which are used to evaluate the risk of DS consist of measuring combinations of biomarkers in maternal serum, including alpha fetoprotein (AFP), total human chorionic gonadotropin (hCG), free beta subunit of hCG (β-hCG), unconjugated estriol (uE3), pregnancy-associated plasma protein A (PAPP-A), proform of eosinophil major basic protein (ProMBP), inhibin-A, and placental growth factor (PGF). Studies of DS-affected pregnancies have demonstrated that serum screening tests exhibit sensitivities of 70–85% and specificities of 90–96%^16^,^17^,^18^. In recent years, ultrasonographic nuchal translucency (NT) measurements during the first trimester of gestation have been used in combination with serum screening tests; the integrated screening tests yield sensitivities of 90–95% with acceptable false-positive (FP) rates^19^,^20^. The development and application of these prenatal screening tests have prevented many unaffected pregnant women from undergoing invasive techniques, such as chorionic villus sampling or amniocentesis, which can cause miscarriages (reported risks of 0.6–2%^21^); additionally, these tests have provided a large number of significant clues for the prevention and diagnosis of DS-affected pregnancies^22^.

The serum triple screening test (STS) composed of AFP, uE3, and hCG (or β-hCG) measurements during the second trimester is one of the most commonly used prenatal screening tools for DS, although the sensitivity and specificity of this test are unsatisfactory^23^,^24^. The integrated screening test (INS), which consists of an NT measurement and various serum biochemical marker screening tests during the first or second trimesters, exhibits a markedly improved sensitivity, with relatively few FPs^20^. However, there are large disparities in the sensitivity and specificity of the STS among different studies, with sensitivities ranging from 69%^24^ to 92%^25^ and specificities ranging from 81%^26^ to 96%^17^. Additionally, significant differences in the DS detection rate of the STS and INS tests have been reported. Thus, to systematically assess the diagnostic value of the STS and INS for the prenatal screening of DS and to evaluate the differences in screening results between the two tests, a systematic review and meta-analysis of relevant studies was performed, and meta-regression analyses and a funnel plot asymmetry test were conducted to explore the sources of heterogeneity and to evaluate the risk of bias across the included studies using Meta-Disc (version 1.4) and Rev Man (version 5.2) software, respectively.

## Results

### Study selection

**Figure 1:**
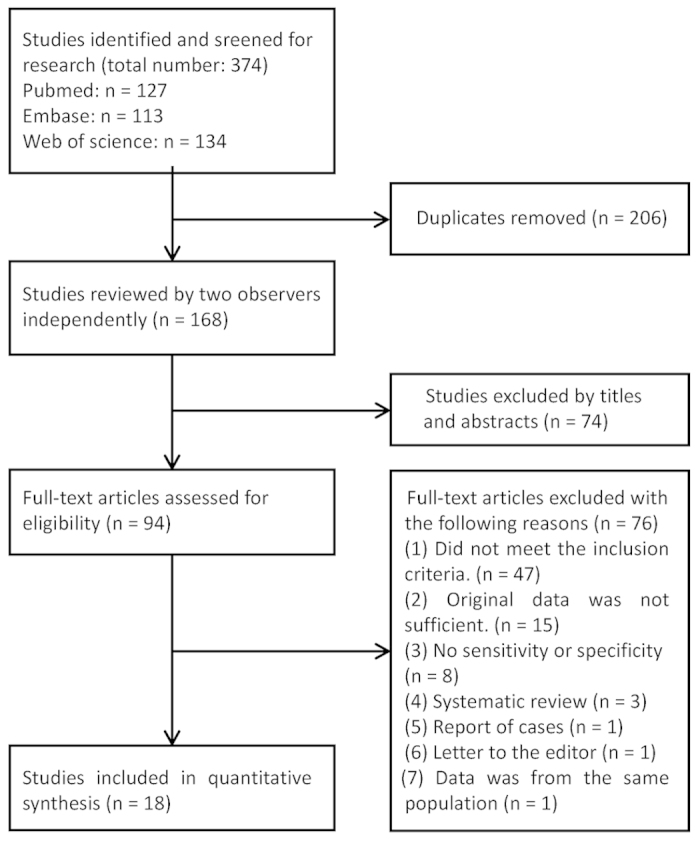
Flowchart of the literature search and study selection.

A total of 374 records were initially identified from various databases and sources; 206 of these publications were excluded because they contained duplicate data. The remaining 168 articles were screened by two independent observers (Y.Y. and Y.L.). Seventy-four studies were excluded based on the title and abstract, and 94 full-text articles were considered relevant and were further examined in detail. Seventy-six of these papers were subsequently excluded for the reasons presented in Fig. 1. Finally, a total of 18 articles (13 related to the STS and 6 related to the INS, and one of these articles used both tests) met the selection criteria and were analysed in the meta-analysis.

### Study characteristics and analysis of results

The characteristics of the studies are presented in Table 1. A total of 18 articles^10^,^16^,^17^,^18^,^19^,^20^,^23^,^24^,^25^,^26^,^27^,^28^,^29^,^30^,^31^,^32^,^33^,^34^ with 183,998 samples were included in this meta-analysis. The sample sizes of these studies ranged from 221 to 37,362, and the average number of samples per study was 10,222. The articles were published between September 1997^26^ and October 2012^18^. Seven studies were from European countries, seven were from Asian countries, and four were from North and South American countries, including the USA and Venezuela. All of the publications were original research articles, with the exception of a meeting report published by Smetanova *et al.* in 2009. All of the specimens consisted of maternal serum; twelve studies obtained serum during the second trimester, and the remaining studies obtained serum during both the first and second trimesters. Most studies used the test consisting of AFP, uE3, and total hCG (n = 12); some studies used the AFP, uE3, and β-hCG combination (n = 4); and both of these biomarker combinations were used in the remaining two studies. Two-thirds of the articles performed prenatal screening without an NT measurement (n = 12); the other studies used the INS test, which included NT measurements, the STS and other maternal serum biomarkers, such as PAPP-A and inhibin-A (n = 6). One of the studies used both the STS and INS. The STS was primarily conducted during the second trimester of pregnancy, whereas NT measurements were performed between 10 and 14 weeks of gestation or during the first trimester. The quality of the studies was also assessed by three independent reviewers (Y.Y., M.H., and B.Z.) using the Quality Assessment of Diagnostic Accuracy Studies (QUADAS-2) criteria. The quality scores of these 18 studies ranged from 8 to 13 (Table 1). The scores of 13 studies were greater than 9, and 1 and 4 studies had quality scores of 8 and 9, respectively (see Table 1 in supplementary information). Risk thresholds, which were not reported in some articles, were set between 1/250 and 1/300 in most of the articles. All of the studies reported true positive (TP), true negative (TN), FP, and false negative (FN) values, which are presented in Table 1.

### Diagnostic performance

Spearman correlation coefficients for the STS and INS were 0.505 (p = 0.078) and 0.429 (p = 0.397), respectively, and no threshold effect was observed. The data extracted from the studies were integrated to produce a pooled sensitivity, pooled specificity, pooled positive likelihood ratio (PLR), pooled negative likelihood ratio (NLR), and pooled diagnostic odds ratio (DOR).

**Figure 2:**
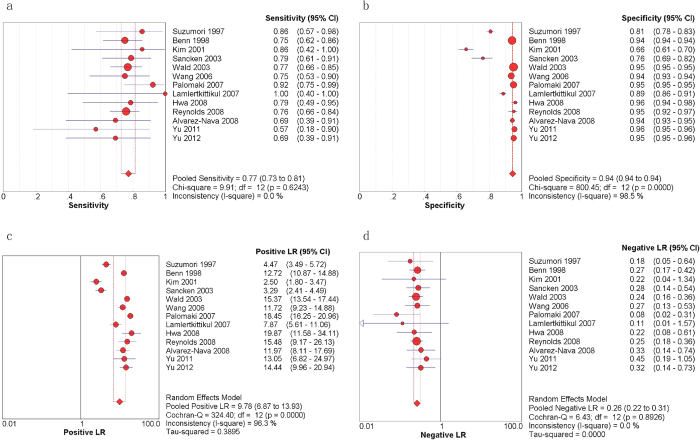
Forest plots estimating the sensitivity (a), specificity (b), PLR (c), and NLR (d) of the STS analysis with 95% CIs. Each red solid circle represents a study, and the size of the red circle indicates the number of samples in each study. Error bars denote the 95% CI.

**Figure 3:**
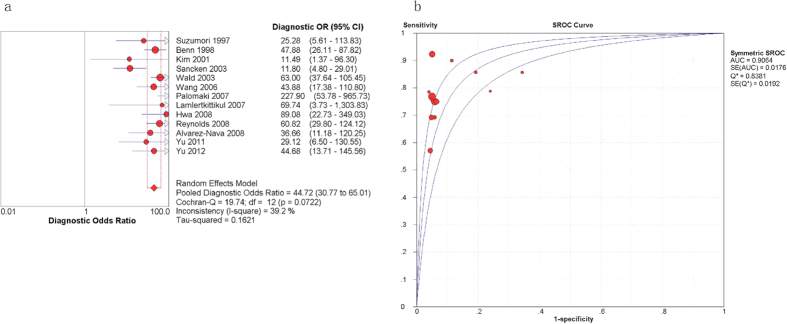
Forest plots estimating the DOR (a) and SROC curves (b) of the STS analysis. Each red solid circle represents a study, and the size of the red circle indicates the number of samples in each study. Error bars denote the 95% CI.

The results of the sensitivity, specificity, PLR, and NLR for the STS are presented in Fig. 2. The sensitivities ranged from 0.57 and 1.00 in the 13 relevant articles, and the pooled sensitivity was 0.77 with a 95% confidence interval (CI) of 0.73–0.81 (Fig. 2a). The specificities ranged from 0.66 to 0.96, and the pooled specificity was 0.94 with a 95% CI of 0.94–0.94 (Fig. 2b). The PLR and NLR were 9.78 (95% CI = 6.87–13.93) (Fig. 2c) and 0.26 (95% CI = 0.22–0.31) (Fig. 2d), respectively. Figure 3 presents the DOR and the summary receiver operating characteristic (SROC) curve that was used to evaluate the relationship between the sensitivity and specificity across all of the 13 studies. The pooled DOR was 44.72 with a 95% CI of 30.77–65.01 (Fig. 3a). The Q value of the SROC curve was 0.8381, and the area under the curve (AUC) was 0.9064 (Fig. 3b). The heterogeneity of the results was assessed using the inconsistency (I-square) test and the chi-square or Cochran-Q test; significant heterogeneity was found in the specificity (p = 0.00, I-square = 98.5%), PLR (p = 0.00, I-square = 96.3%), and DOR (p = 0.07, I-square = 39.2%).

**Figure 4:**
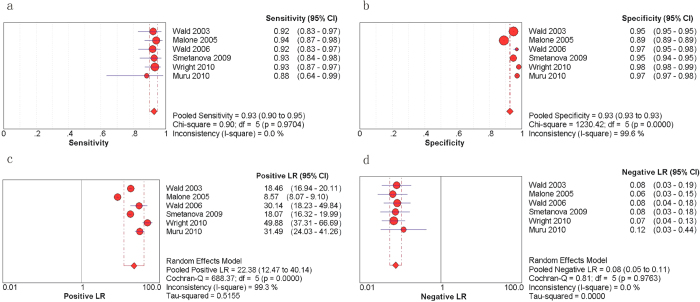
Forest plots estimating the sensitivity (a), specificity (b), PLR (c), and NLR (d) of the INS analysis with 95% CIs. Each red solid circle represents a study, and the size of the red circle indicates the number of samples in each study. Error bars denote the 95% CI.

**Figure 5:**
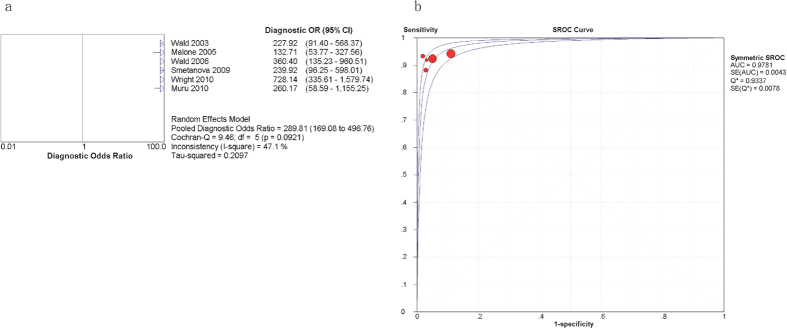
Forest plots estimating the DOR (a) and SROC curves (b) of the INS analysis. Each red solid circle represents a study, and the size of the red circle indicates the number of samples in each study. Error bars denote the 95% CI.

The diagnostic performance of the INS is described in Figs 4 and 5. In the six applicable studies, the sensitivities ranged from 0.88 to 0.94, and the pooled sensitivity was 0.93 with a 95% CI of 0.90–0.95 (Fig. 4a). The specificities ranged from 0.89 to 0.98, and the pooled specificity was 0.93 with a 95% CI of 0.93–0.93 (Fig. 4b). The PLR and NLR were 22.38 (95% CI = 12.47–40.14) (Fig. 4c) and 0.08 (95% CI = 0.05–0.11) (Fig. 4d), respectively. As presented in Fig. 5, the pooled DOR was 289.81, with a 95% CI of 169.08–496.76. The Q value and AUC for the SROC curve were 0.9337 and 0.9781, respectively. The chi-square and inconsistency tests indicated significant heterogeneity in the specificity (p = 0.00, I-square = 99.6%), PLR (p = 0.00, I-square = 99.3%), and DOR (p = 0.09, I-square = 47.1%) of the INS.

**Figure 6:**
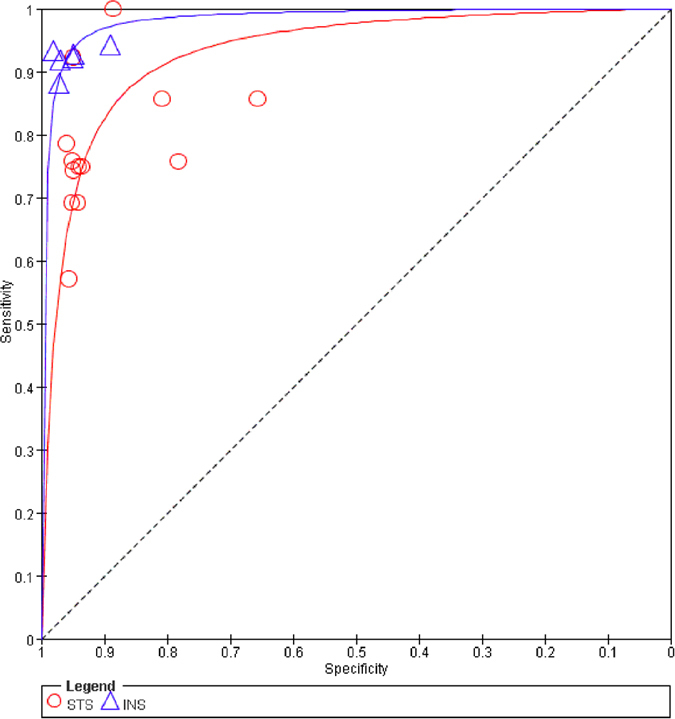
SROC curves for both the STS and INS. The red circles represent the studies included in the STS analysis, and the blue triangles represent the studies included in the INS analysis.

To compare the inherent diagnostic performance of the STS and INS, the two SROC curves were superimposed (Fig. 6). The INS curve was above the STS curve. A z test comparing the AUC and Q values for both tests indicated that both of these parameters were higher for the INS than for the STS (z statistic = 3.957, p < 0.01; z statistic = 4.613, p < 0.01, respectively) (see Tables 4 and 5 in supplementary information).

### Regression analysis and evaluation of publication bias

**Figure 7:**
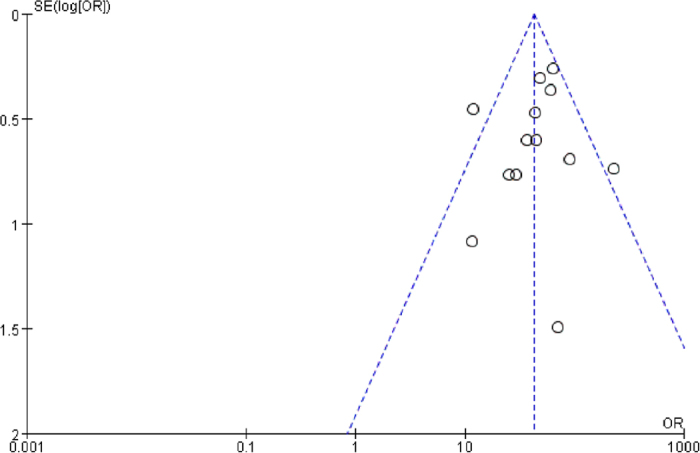
Funnel plot indicating no significant publication bias in the STS analysis.

Multivariate and univariate meta-regression analyses were conducted to explore the sources of heterogeneity in the specificity, PLR, and DOR. The covariate design, blinding, country/region, total number of specimens (TNS), combination modes used for the prenatal screening test (CMPST), and quality were examined in this analysis. The results of the multivariate regression analyses for the STS and INS are presented in Tables 2 and 3, respectively; no statistical significance in the relative DOR (RDOR) values across studies was found for either test. Next, a univariate meta-regression analysis for each covariate revealed no significant differences between the two tests. A funnel plot asymmetry test was performed using Rev Man 5.2 to evaluate the risk of publication bias in the articles pertaining to the STS (Fig. 7). The overall distribution of the study points was symmetric, and the inverted funnel shape of the plot suggests that there was no significant publication bias in the STS analysis.

## Discussion

Non-invasive prenatal risk assessments for the most common aneuploidies are typically offered before resorting to invasive prenatal procedures. Various biochemical screening tests of maternal serum are performed during the first or second trimesters along with ultrasonographic NT measurements. These screening tests have greatly improved prenatal screening for DS-affected pregnancies; however, there are obvious discrepancies in the diagnostic performance of these two tests. In this meta-analysis, 18 articles were finally examined, one of which was included in both screening tests, and 13 and 6 studies were analysed to evaluate the performance of the STS and INS in prenatal DS screening, respectively. The QUADAS-2 tool was used to assess the quality of these studies, and most of the studies had scores of 10 or more, indicating relatively high quality.

Our study indicates that the respective pooled sensitivities and specificities were 0.77 (95% CI = 0.73–0.81) and 0.94 (95% CI = 0.94–0.94) for the STS and 0.93 (95% CI = 0.90–0.95) and 0.93 (95% CI = 0.93–0.93) for the INS. The respective AUC and Q values for the SROC curves were 0.9064 and 0.8381 for the STS and 0.9781 and 0.9337 for the INS. These results indicate that both tests exhibit a high level of accuracy and that they are effective screening tools for DS. Moreover, the INS curve was higher than the STS curve, and the AUC and Q values for the INS were greater than the STS values (p < 0.01 for both). This finding suggests that the INS markedly outperformed the STS. The pooled sensitivity of the INS was greater than that of the STS in our study, and there was no overlap in the 95% CIs of the two tests, indicating that the former was more sensitive than the latter. However, there was no significant difference in specificity. Thus, the superior diagnostic performance of the INS can largely be attributed to its higher sensitivity. The DOR is another indicator of test performance^35^ that is derived from the integration of the sensitivity and specificity data and presents the ratio of the odds of experiencing positive test results in a diseased population to the odds of experiencing a positive test result in a non-diseased population^36^. DOR values are indicators of the diagnostic performance of prenatal screening tests, with greater values indicating better test performance^37^ and values equal to or less than 1 indicating no ability to diagnose DS-affected pregnancies. In the present meta-analysis, the pooled DOR values were 44.72 (95% CI = 30.77–65.01) for the STS and 289.81 (95% CI = 169.08–496.76) for the INS, further suggesting that the INS exhibits a better diagnostic performance than the STS. In comparison to the DOR and SROC, likelihood ratios are considered to have greater clinical value^38^. The PLR and NLR for the STS were 9.78 and 0.26, respectively. Thus, the ratio of the probability of having a positive test result to the probability of having a negative test result was 9.78 in patients with DS and 0.26 in unaffected patients. For the INS, the PLR and NLR were 22.38 and 0.08, respectively, indicating that the INS was better able to discriminate between DS-affected and unaffected patients.

Significant heterogeneity was identified in the specificity, PLR, and DOR results of the two tests. Because the exploration of sources of heterogeneity is an important part of any meta-analysis^36^, we conducted an analysis of diagnostic thresholds and meta-regression analyses. The results from the analysis of diagnostic thresholds indicated that there was no threshold effect for either test, suggesting that the heterogeneity results from a non-threshold effect in this study. Thus, a multivariate meta-regression analysis with six covariates and univariate meta-regression analyses for each covariate were performed. None of the covariates were found to be responsible for the significant heterogeneity. Recent studies have reported that the inclusion of different populations and the selection of different study designs may influence the diagnostic accuracy of a test^39^,^40^. The discrepancies in heterogeneity between these studies and our study might be due to differences in sample size^40^, biochemical markers^41^, and/or pathological states of the affected populations^39^. Moreover, because significant heterogeneity was found largely in the specificity and PLR of our study and others^37^,^41^, we inferred that risk thresholds might be another cause of the heterogeneity. However, we could not include these covariates in our meta-regression analysis because of insufficient data in the evaluated studies.

Biases are innate in meta-analyses and can emerge during the course of study selection, integration, and data analysis. Publication bias is one of the most important biases that greatly influences the authenticity and reliability of the meta-analysis results. The funnel plot asymmetry test is the most common method of evaluating publication bias. This test is based on the hypothesis that detection precision improves as sample size increases and that the width of the funnel plot narrows with improvements in detection precision. In funnel plots, studies with small sample sizes often exhibit greater variation and are distributed at the bottom of the plot, whereas larger studies exhibit better precision and are distributed at the top of the plots. As sample sizes increase, the studies cluster near the middle vertical line, the overall distribution of the data points becomes more symmetric, and the plot resembles an inverted funnel. Asymmetry or gaps in the funnel plot are indicative of significant publication bias in the meta-analysis, with the degree of asymmetry reflecting the extent of the bias. In our study, we performed the funnel plot asymmetry test using Rev Man 5.2. The overall distribution of data points was symmetric, and the plot resembled an inverted funnel, suggesting that there was no significant publication bias. Because it can be difficult to evaluate the symmetry of a plot with few studies, the funnel plot should be composed of at least 10 independent studies^42^. Thus, we did not perform the funnel plot asymmetry test for the INS test.

In our study, the pooled sensitivity, pooled DOR, AUC, and Q values of the SROC curve of the INS test were markedly greater than those of the STS test, and the NLR of the INS test was lower than that of the STS, indicating that the INS test exhibits a better diagnostic performance than the STS test. However, both tests yielded specificities of 93–95%, with no significant differences between them, suggesting that the INS did not produce fewer FP results. The relatively low specificity of prenatal screening tests means that many FPs are diagnosed. Consequently, some pregnant women without DS-affected pregnancies may be subjected to further invasive diagnostic procedures that carry a risk of inducing miscarriage^43^. With the current FP rate, approximately 180–190 of the expected 200 patients with DS-affected pregnancies among every 100,000 women who undergo the INS will be identified, and an additional 5,000 unaffected pregnant women will be considered to be at an increased risk and subjected to invasive procedures such as amniocentesis or chorionic villus sampling that can lead to miscarriage. Hence, the diagnostic performance (and specificity in particular) of these prenatal screening tests must be further improved.

As the primary methods of screening for DS are based on a combination of biochemical markers in maternal serum and ultrasound measurements, it is necessary to search for novel predictive biomarkers of DS-affected pregnancies and to then integrate them into more specific and sensitive prenatal screening tests that will improve our ability to accurately diagnose DS^11^. In recent studies, levels of biochemical markers such as complement factor H, Transthyretin^6^, complement factor B, alpha-1B-glycoprotein^44^, arylsulfatase A^5^, and apolipoprotein E^45^ were shown to be altered in maternal serum from women with DS-affected pregnancies. These biomarkers might have diagnostic value. Our previous study demonstrated that the four proteins dGTPase, beta2-glycoprotein I (β2-GPI), complement factor H-related protein 1 precursor (CFHR1), and kininogen 1 isoform 2 are potential predictive biomarkers of DS^46^. In this previous study, we utilised comparative proteomic techniques and western blotting to identify and verify these four potential markers. We found that β2-GPI levels were significantly decreased in maternal serum from women with DS-affected pregnancies and that the levels of the other markers were elevated. These findings are in accordance with the results of other studies^6^,^44^,^47^,^48^. However, further studies are being conducted to validate the abovementioned results in larger populations and to investigate whether these maternal serum biomarkers could be used independently in prenatal screening or used alongside current biomarkers such as AFP, hCG, and uE3 to screen for DS.

Our meta-analysis had several strengths. First, we performed a comprehensive and systematic literature search with appropriate inclusion criteria and no language restrictions, and we screened the references of the identified publications for additional eligible studies. Second, the publication selection and data extraction were conducted independently by two authors, and a third reviewer was involved when discrepancies emerged. Third, the quality of the studies was assessed by three independent reviewers using the QUADAS-2 criteria, and most of the studies were found to have relatively satisfactory quality. Fourth, likelihood ratios were used in the meta-analysis. The PLRs and NLRs are used to estimate the probabilities of affected subjects occurring among the whole population with positive or negative test results, respectively, and are capable of indicating the degree of abnormality for particular detection results without being influenced by the prevalence rates of the disease. Fifth, we performed meta-regression analyses to investigate the sources of heterogeneity and conducted a publication bias analysis to estimate the effect of each individual study on the pooled results. Our study also had some limitations. First, the source of the heterogeneity was not identified despite the fact that we performed multivariate and univariate meta-regression analyses. Second, although eighteen articles were included in our meta-analysis, the number of studies associated with each test was relatively small, especially for the INS (six studies). Finally, the studies were published between 1997 and 2012; this relatively long time span might have decreased the accuracy of the screening results.

Despite these limitations, the results of this meta-analysis indicated that the INS was a more effective screening method than the STS, with a sensitivity of 93% and a specificity of 93%. Therefore, the INS should be recommended as a first-choice screening test for DS. However, further research is needed to identify other biomarkers with higher specificity and more predictive power to improve prenatal screening tests for DS-affected pregnancies.

## Methods

### Search strategy

A systematic literature search was conducted in PubMed (http://www.ncbi.nlm.nih.gov/pubmed), EMBASE (http://www.embase.com/home), and the ISI Science Citation Index (http://apps.isiknowledge.com) using the following keywords and terms: “Down’s syndrome”, “trisomy 21”, “prenatal screening”, “AFP”, “hCG”, “β-hCG”, “uE3”, “NT”, “alpha fetoprotein”, “human chorionic gonadotropin”, “free beta human chorionic gonadotropin”, “unconjugated estriol”, and “nuchal translucency”. The last search was performed in October 2014. The literature search was restricted to human research, and there were no limitations on the language of the articles or the publication type. The reference lists of the retrieved publications were manually checked for further eligible studies.

### Publication selection criteria

The inclusion criteria for studies in this meta-analysis were as follows: (1) the topic of the publication was the study of prenatal screening for DS-affected pregnancies in humans; (2) the studied specimens were maternal serum or plasma; (3) sufficient information was available to calculate the TP, FP, FN, and TN values; (4) only the STS, which included the measurement of AFP, uE3, and hCG (or β-hCG) levels, was used in the studies without NT measurements; (5) in studies with NT measurements, the detection of maternal serum biomarkers including AFP, uE3, and hCG (or β-hCG) was combined with NT measurements, to evaluate the diagnostic performance of the INS; and (6) eligible unpublished data were presented at international meetings. The exclusion criteria were as follows: (1) articles without original data; (2) single case reports; (3) studies in which DS was diagnosed along with other diseases; (4) studies in which DS-affected pregnant women were affected by other factors; and (5) studies without NT measurements in which the STS was conducted along with the detection of other maternal serum biomarkers. In addition to these criteria, all of the articles were carefully analysed and compared to ensure that no duplicate reports from the same patient population were included in the meta-analysis.

### Data extraction

Relevant information, including names of first authors, publication years, countries where the studies were performed, sources and types of samples, total numbers of samples, numbers of samples with the determined test results (TP, TN, FP, FN), CMPSTs, risk thresholds for prenatal screening of DS, and QUADAS scores, was carefully extracted from all of the selected studies. Study data were independently extracted from each article by two of the authors (Y.Y. and Y.L.), and a third author was involved (B.Z.) when discrepancies arose.

### Quality assessment of selected studies

The quality of each study was assessed by three independent reviewers (Y.Y., M.H., and B.Z.) using the QUADAS-2 criteria for the quality assessment of diagnostic accuracy studies^49^.

### Data processing and statistical analysis

A meta-analysis of the diagnostic test studies was conducted to evaluate the performance of the prenatal screening test in each study using Meta-Disc (version 1.4) and Rev Man (version 5.2) software^50^. The TP, TN, FP, and FN data were extracted from the selected studies, and the analysis of diagnostic thresholds was performed to determine whether a diagnostic threshold effect was present. Spearman correlation coefficients were used in this analysis, and a positive correlation indicated the existence of a threshold effect (p < 0.05)^37^. If the threshold effect existed, the extracted data were pooled by fitting SROC curves, and the AUC was calculated for each curve. If no threshold effect was present, further pooling was performed.

The heterogeneity of the included studies was evaluated using the chi-square test (Cochran-Q statistic) and the inconsistency measure (I-squared statistic). A p value < 0.10 in the Cochran-Q test or an I-squared value > 30% indicated significant heterogeneity^37^. Random-effects or fixed-effects models for the meta-analysis were used according to the results of heterogeneity analysis. The pooled sensitivity, specificity, PLR, NLR, and DOR with 95% CIs were calculated for all of the selected studies, and the SROC curves for the STS and INS tests were produced using Meta-Disc (version 1.4) software. To evaluate the differences in the diagnostic performance of the two tests, the two SROC curves were superimposed using Rev Man 5.2, and a z test was performed to compare the AUC of the two curves.

### Meta-regression analysis and evaluation of publication bias

When significant heterogeneity was found, the sources of heterogeneity were explored using multivariate meta-regression analyses with Meta-Disc (version 1.4) with several covariates, including study design, blinding, country/region, TNS, CMPST, and quality. A univariate regression analysis was also performed for each covariate if no significant difference was found in the multivariate meta-regression analyses. The covariates were considered to have a statistically significant effect in the meta-regression models if p < 0.05. Finally, a funnel plot asymmetry test was conducted using Rev Man 5.2 to estimate the risk of bias across the articles included in the meta-analysis.

## Additional Information

**How to cite this article**: Yao, Y. *et al.* Two kinds of common prenatal screening tests for Down’s syndrome: a systematic review and meta-analysis. *Sci. Rep.* **6**, 18866; doi: 10.1038/srep18866 (2016).

## Supplementary Material

Supplementary Information

## Figures and Tables

**Table 1:** Characteristics of the 18 studies included in the meta-analysis.

Author/Year	Country/Region	TOA	TOS	Gestational Age	CMPST	QS	TNS	TP	FP	FN	TN	Risk Threshold
Suzumori, 1997	Japan	OR	MS	15–18 wks	STST	10	1067	12	202	2	851	1/299
Benn, 1998	USA	OR	MS	ST	STST	12	34368	42	2023	14	32289	1/270
Kim, 2001	Korea	OR	MS	ST	STST	11	453	6	153	1	293	1/270
Sancken, 2003	Deutschland	OR	MS	ST	STST or STSB	9	221	26	45	7	143	NA
Wald, 2003	UK	OR	MS	10, 1–20 wks	NT+hCG+PAPP-A+STST/STSB	11	37362	60	1516	5	28794	NA
Malone, 2005	USA	OR	MS	10–14, 15–18 wks	NT+β-hCG+PAPP-A+Inhibin A+STST	13	33546	82	3680	5	29779	1/250 (FT) 1/300 (ST)
Wang, 2006	China	OR	MS	ST	STSB	11	15120	18	966	6	14130	1/270
Wald, 2006	UK	OR	MS	10–13, 14–22 wks	NT+PAPP-A+STST	9	566	68	15	6	477	1/300
Palomaki, 2007	USA	OR	MS	ST	STST	9	18898	24	944	2	17928	NA
Lamlertkittikul, 2007	Thailand	OR	MS	14–20 wks	STST	10	996	4	113	0	879	1/250
Reynolds, 2008	UK	OR		ST	STST	10	381	72	14	23	272	1/250
Hwa, 2008	Taiwan	OR	MS	ST	STST	9	444	11	17	3	413	1/270
Alvarez-Nava, 2008	Venezuela	OR	MS	15–20 wks	STSB	12	3005	9	173	4	2819	1/270
Smetanova, 2009	Czech	MR	MS	FT, ST	NT+β-hCG+PAPP-A+STST	8	11743	65	600	5	11073	NA
Wright, 2010	UK	OR	MS	10–14, 15–19 wks	NT+β-hCG+PAPP-A+inhibin A+STST	11	2579	111	46	8	2414	1/200
Muru, 2010	Estonia	OR	MS	FT, ST	NT+β-hCG+PAPP-A+STST	12	3122	15	87	2	3018	1/50 (FT) 1/270 (ST)
Yu, 2011	China	OR	MS	ST	STSB	10	9143	4	400	3	8736	1/270
Yu, 2012	China	OR	MS	ST	STSB	11	10984	9	526	4	10445	1/270

TOA: type of article; OR: original research; MR: meeting report; TOS: type of specimen; MS: maternal serum; wks: weeks; ST: second trimester; FT: first trimester; CMPST: combination modes used for prenatal screening tests; STST: serum triple screening test comprising AFP, uE3, and total hCG; STSB: serum triple screening test comprising AFP, uE3, and β-hCG; QS: QUADAS score; TNS: total number of specimens; TP: true positive; FP: false positive; FN: false negative; TN: true negative; NA: not available (see Tables 2 and 3 in supplementary information).

**Table 2:** Results of the backward meta-regression analysis for the most important covariates for the STS test (inverse variance weights).

Variable	Coeff.	Std. Err.	p-value	RDOR	[95% CI]
Cte.	4.263	0.8639	0.0043	—	—
S	−0.120	0.2266	0.6200	—	—
Design	0.685	0.4941	0.2243	1.98	(0.56; 7.07)
Blindedness	−1.601	0.9809	0.1636	0.20	(0.02; 2.51)
Country/Region	0.361	0.3943	0.4023	1.43	(0.52; 3.95)
TNS	0.683	0.4838	0.2173	1.98	(0.57; 6.86)
CMPST	−0.180	0.3845	0.6602	0.84	(0.31; 2.25)
Quality	−0.351	0.4935	0.5086	0.70	(0.20; 2.50)

RDOR: Relative diagnostic odds ratio; Cte: Constant coefficient; S: Statistic S; TNS: total number of specimens; CMPST: combination modes used for prenatal screening tests.

**Table 3:** Results of the backward meta-regression analysis for the most important covariates for the INS test (inverse variance weights).

Variable	Coeff.	Std. Err.	p-value	RDOR	[95% CI]
Cte.	6.380	1.0548	0.1043	—	—
S	−0.086	0.7303	0.9250	—	—
Design	−0.377	0.6701	0.6738	0.69	(0.00; 3420.26)
Blindedness	−1.054	0.7386	0.3890	0.35	(0.00; 4149.18)
Country/Region	0.472	1.0911	0.7401	1.60	(0.00; 1681999.23)
Cte.	4.668	1.1196	0.1499	—	—
S	−0.663	0.5114	0.4182	—	—
TNS	−0.193	0.7297	0.8353	0.82	(0.00; 8770.69)
CMPST	0.432	0.4255	0.4955	1.54	(0.01; 343.33)
Quality	−0.359	0.6182	0.6652	0.70	(0.00; 1801.63)

RDOR: Relative diagnostic odds ratio; Cte: Constant coefficient; S: Statistic S; TNS: total number of specimens; CMPST: combination modes used for prenatal screening tests.
