# Supplementary material for: Two kinds of common prenatal screening tests for Down’s syndrome: a systematic review and meta-analysis
Source: Sci Rep. 2016 Jan 6;6:18866. doi: 10.1038/srep18866 (PMC4702166; doi:10.1038/srep18866)
Supplement: Supplementary Information [file srep18866-s1.pdf]

Manuscript title: Two kinds of common prenatal screening tests for Down's syndrome: a systematic review and meta-analysis

Author and Affiliation: Yuan Yao[1, 2], Yang Liao[3], Mei Han[1], Sheng-Lan Li[1], Juan Luo[1] & Bo Zhang[1]*

[1]Department of Laboratory Medicine, Southwest Hospital, Third Military Medical University of PLA, Chongqing 400038, PR China.

[2]Department of Laboratory Medicine, No. 191 Clinical Department of No. 303 Hospital of PLA, Guigang 537100, Guangxi, PR China.

[3]Department of Laboratory Medicine, Guangzhou General Hospital of Guangzhou Military Command of PLA, Guangzhou 510010, Guangdong, PR China.

*Correspondence and requests for materials should be addressed to B.Z. (zhangbocq@aliyun.com.)

Supplementary Information for the manuscript (SREP-15-01307B)

Table 1 | Results of quality assessment of selected studies using the QUADAS-2 criteria

Author/Year	RT	1st	2nd	3rd	4th	5th	6th	7th	8th	9th	10th	11th	12th	13th	14th	TS
**Suzumori, 1997**	1/299	0	0	1	1	1	1	1	1	1	1	1	0	0	1	10
**Benn, 1998**	1/270	1	1	1	1	1	1	1	1	0	1	1	0	1	1	12
**Kim, 2001**	1/270	0	0	1	1	1	1	1	1	1	1	1	1	0	1	11
**Sancken, 2003**	NA	0	0	1	1	1	1	1	1	0	1	1	1	0	0	9
**Wald, 2003**	NA	1	1	1	1	1	1	1	1	0	1	1	1	0	0	11
**Malone, 2005**	1/250 (FT)<br>1/300 (ST)	1	1	1	1	1	1	1	1	1	1	1	1	0	1	13
**Wang, 2006**	1/270	1	1	1	1	1	1	1	1	0	1	1	1	0	0	11
**Wald, 2006**	1/300	0	1	1	1	1	1	1	1	0	1	1	0	0	0	9
**Palomaki, 2007**	NA	1	0	1	1	1	1	1	1	0	1	1	0	0	0	9
**Lamlertkittikul, 2007**	1/250	0	1	1	1	1	1	1	1	0	1	1	0	0	1	10
**Reynolds, 2008**	1/250	0	1	1	1	1	1	1	1	0	1	1	1	0	0	10
**Hwa, 2008**	1/270	0	1	1	1	1	1	1	1	0	1	1	0	0	0	9
**Alvarez-Nava, 2008**	1/270	1	1	1	1	1	1	1	1	1	1	1	0	0	1	12
**Smetanova, 2009**	NA	1	0	1	1	1	1	1	0	0	1	1	0	0	0	8
**Wright, 2010**	1/200	0	1	1	1	1	1	1	1	0	1	1	1	0	1	11
**Muru, 2010**	1/50 (FT)<br>1/270 (ST)	0	1	1	1	1	1	1	1	1	1	1	1	0	1	12
**Yu, 2011**	1/270	1	1	1	1	1	1	1	1	0	1	1	0	0	0	10
**Yu, 2012**	1/270	1	1	1	1	1	1	1	1	0	1	1	1	0	0	11

QUADAS: Quality Assessment of Diagnostic Accuracy Studies; RT: Risk Threshold; NA: not available; “1” means “Yes, this study meets the criterion”; “0” means “No, this study does not meet the criterion”; TS: total score.

Table 2 | Primary data of the 13 studies included in the STS

Author/Year	Country/Region	Gestational Age	TNS	TP	FP	FN	TN	Risk Threshold
**Suzumori, 1997**	Japan	15-18 wks	1067	12	202	2	851	1/299
**Benn, 1998**	USA	ST	34368	42	2023	14	32289	1/270
**Kim, 2001**	Korea	ST	453	6	153	1	293	1/270
**Sancken, 2003**	Deutschland	ST	221	26	45	7	143	NA
**Wald, 2003**	UK	10, 14-20 wks	37362	60	1516	5	28794	NA
**Wang, 2006**	China	ST	15120	18	966	6	14130	1/270
**Palomaki, 2007**	USA	ST	18898	24	944	2	17928	NA
**Lamlertkittikul, 2007**	Thailand	14-20 wks	996	4	113	0	879	1/250
**Reynolds, 2008**	UK	ST	381	72	14	23	272	1/250
**Hwa, 2008**	Taiwan	ST	444	11	17	3	413	1/270
**Alvarez-Nava, 2008**	Venezuela	15-20 wks	3005	9	173	4	2819	1/270
**Yu, 2011**	China	ST	9143	4	400	3	8736	1/270
**Yu, 2012**	China	ST	10984	9	526	4	10445	1/270

ST: second trimester; wks: weeks; TNS: total number of specimens; TP: true positive; FP: false positive; FN: false negative; TN: true negative; NA: not available.

Table 3 | Primary data of the 6 studies included in the INS

Author/Year	Country/Region	Gestational Age	TNS	TP	FP	FN	TN	Risk Threshold
**Wald, 2003**	UK	10, 14-20 wks	37362	60	1516	5	28794	NA
**Malone, 2005**	USA	10-14, 15-18 wks	33546	82	3680	5	29779	1/250 (FT)<br>1/300 (ST)
**Wald, 2006**	UK	10-13, 14-22 wks	566	68	15	6	477	1/300
**Smetanova, 2009**	Czech	FT, ST	11743	65	600	5	11073	NA
**Wright, 2010**	UK	10-14, 15-19 wks	2579	111	46	8	2414	1/200
**Muru, 2010**	Estonia	FT, ST	3122	15	87	2	3018	1/50 (FT)<br>1/270 (ST)

ST: second trimester; FT: first trimester; wks: weeks; TNS: total number of specimens; TP: true positive; FP: false positive; FN: false negative; TN: true negative; NA: not available.

Table 4 | Results of z test between the AUC of STS and INS

Type of study	NIS	AUC	SE of AUC	Z statistic	p value	Significant difference
**INS**	6	0.9781	0.0043	3.957	p =0.0001	Yes
**STS**	13	0.9064	0.0176			

NIS: number of included studies; AUC: area under ROC curve; SE: standard error.

Table 5 | Results of z test between the Q values of STS and INS

Type of study	NIS	AUC	SE of AUC	Z statistic	p value	Significant difference
**INS**	6	0.9337	0.0078	4.613	P<0.0001	Yes
**STS**	13	0.8381	0.0192			

NIS: number of included studies; AUC: area under ROC curve; SE: standard error.
